# Supplementary material for: Incidental Diagnosis of Urothelial Bladder Cancer: Associations with Overall Survival
Source: Cancers (Basel). 2023 Jan 21;15(3):668. doi: 10.3390/cancers15030668 (PMC9913049; doi:10.3390/cancers15030668)
Supplement: Supplementary file 1 [file cancers-15-00668-s001.zip › cancers-2131814-supplementary.pdf]

Supplementary files:

Table S1. Comparison of included patients and patients excluded due to unknown diagnosis type.

		Excluded due to unknown diagnosis type (n = 63)	Included (n = 435)	p-value
**Primary grade**	LG	37 (59%)	194 (45%)	**0.036**
	HG	26 (41%)	241 (55%)	
Primary stage	NMIBC	53 (84%)	331 (76%)	0.156
	MIBC	6 (10%)	68 (16%)	0.203
	Tx, non-metastatic	3 (5%)	25 (6%)	1.000
	Metastatic	1 (2%)	11 (3%)	1.000

Table S2. Causes of diagnostic evaluation among non-incidentally diagnosed patients.

**Cause of diagnostic evaluation**	**Nonincidental diagnosis (n = 310)**
Gross hematuria	282 (90.9%)
Microscopic hematuria	14 (4.5%)
Hematuria, unknown	1 (0.3%)
Irritative symptoms	10 (3.2%)
Other symptoms	3 (1.0%)

Table S3: Comparison of incidentally diagnosed patients: diagnosed with ultrasound vs diagnosed with other studies.

		Incidental with ultrasound (n = 83)	Other incidental (n = 42)	p-value
**Primary grade**	LG	54 (65%)	23 (55%)	0.263
	HG	29 (35%)	19 (45%)	
Primary stage	NMIBC	74 (89%)	39 (93%)	0.749
	MIBC	6 (7%)	1 (2%)	0.422
	Tx, non-metastatic	3 (4%)	1 (2%)	1.000
	Metastatic	0 (0%)	1 (2%)	0.336

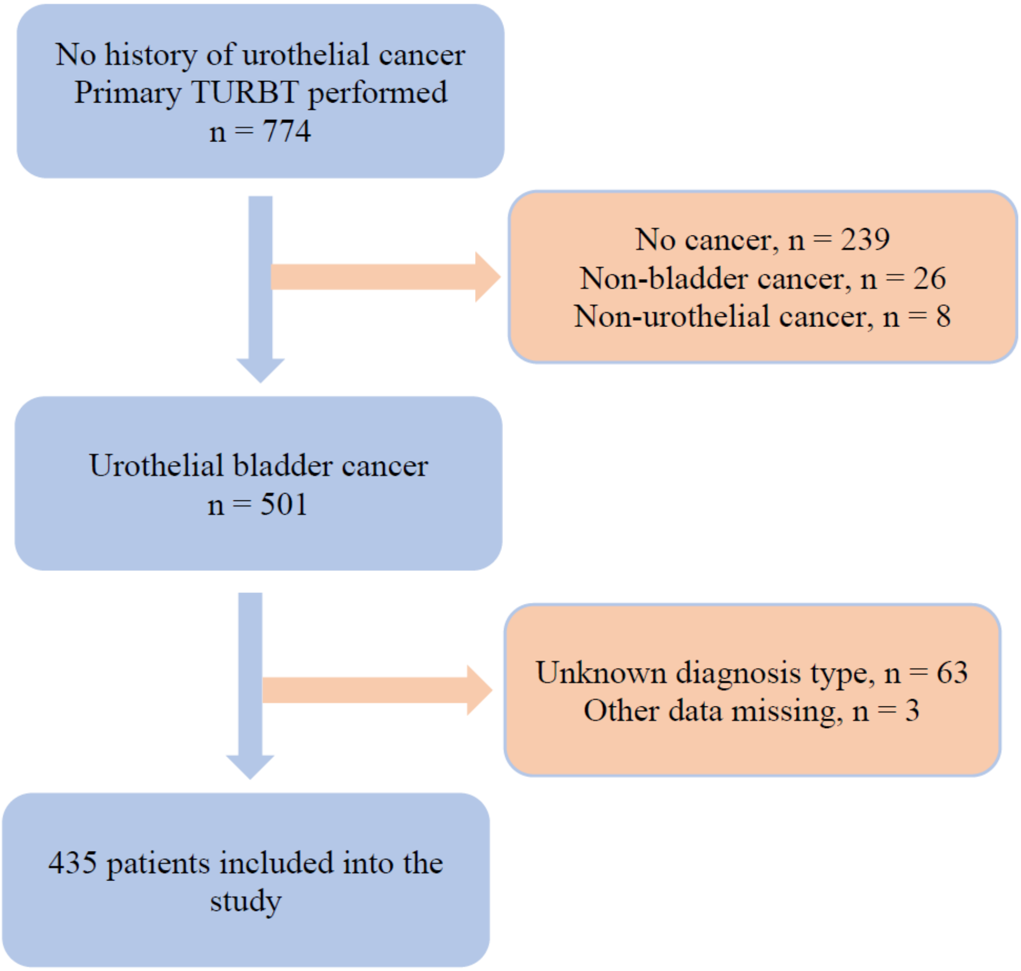

Figure S1. Flowchart of patient inclusion.

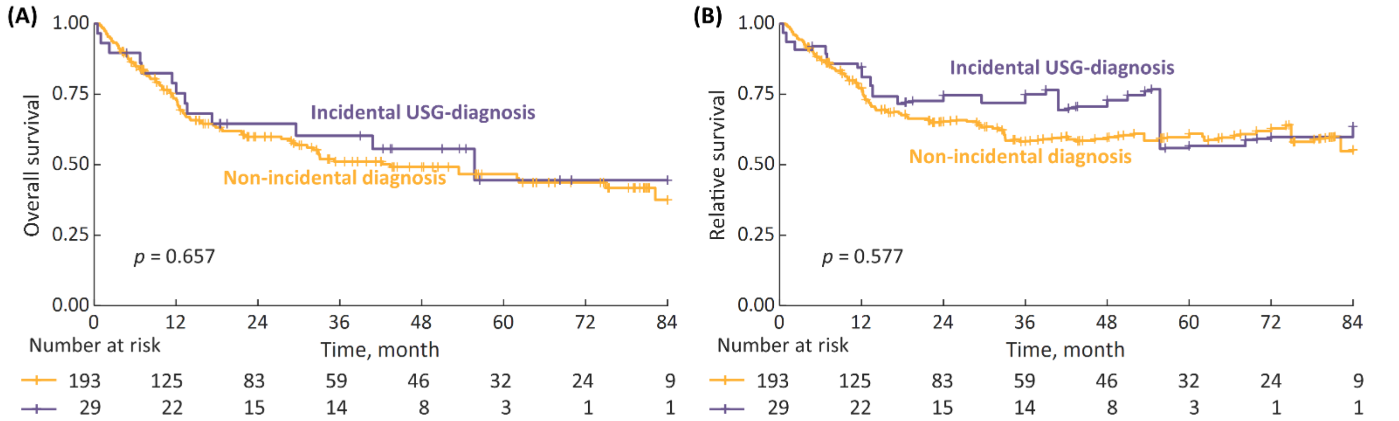

Figure S2. (A) 7-year overall survival (OS) in high-grade (HG) patients stratified by type of diagnosis (in-cidental vs. non-incidental with ultrasound); and (B) 7-year RS.
